# Optimization of Co-Culture Conditions for a Human Vascularized Adipose Tissue Model

**DOI:** 10.3390/bioengineering7030114

**Published:** 2020-09-17

**Authors:** Feipeng Yang, Ronald N. Cohen, Eric M. Brey

**Affiliations:** 1 Department of Biomedical Engineering, Illinois Institute of Technology, Chicago, IL 60616, USA; fyang26@hawk.iit.edu; 2 Department of Medicine, The University of Chicago, Chicago, IL 60637, USA; roncohen@medicine.bsd.uchicago.edu; 3 Department of Biomedical Engineering and Chemical Engineering, University of Texas at San Antonio, San Antonio, TX 78249, USA

**Keywords:** vascularization, adipose tissue, co-culture, microfluidics, adipogenesis

## Abstract

In vitro adipose tissue models can be used to provide insight into fundamental aspects of adipose physiology. These systems may serve as replacements for animal models, which are often poor predictors of obesity and metabolic diseases in humans. Adipose tissue consists of a rich vasculature that is essential to its function. However, the study of endothelial cell–adipocyte interactions has been challenging due to differences in culture conditions required for the survival and function of each cell type. To address this issue, we performed an extensive evaluation of the cell culture media composition to identify the conditions optimal for the co-culture of endothelial cells and adipocytes. The effects of individual media factors on cell survival, proliferation, and differentiation were systematically explored. Several media factors were determined to disrupt the co-culture system. Optimized culture conditions were identified and used to generate a vascularized human adipose microtissue. An interconnected vascular network was established within an adipose micro-tissue, and the networks were anastomosed with perfused channels to form a functional network. In conclusion, media conditions were identified that enabled endothelial cell–adipocyte co-culture and were used to support the formation of a vascularized adipose tissue within a microfluidic device.

## 1. Introduction

Adipose tissue is distributed throughout the body and serves to mechanically protect organs and limbs, as well as playing a vital role in the systemic regulation of metabolism [1]. Poorly functioning adipose tissue is a significant factor contributing to the excess morbidity and mortality observed in healthcare. Obesity alone generates a burden of over $190 billion annually on the American system [2], and globally contributes to over 4.5 million deaths annually [3]. Despite significant research, medical options for the control of adipose expansion and function are limited. Advances in the in vitro models of adipose tissue available for research may allow the improved development of therapeutics and understanding of the underlying mechanisms of disease.

Adipose tissue is one of the most highly vascularized tissues in the body, with each adipocyte in close proximity to one or more capillaries [4]. Adipogenesis and angiogenesis are temporally and spatially coupled in vivo [4]. Vascular remodeling may alter the nutrient and oxygen supply within adipose tissue and influence the number and size of adipocytes, playing an important role in the overall expansion of or reduction in adipose tissue volume. Specifically, adipose tissue may become hypoxic in the setting of obesity, which contributes to metabolic dysfunction [5]. The alteration of vascular function in adipose tissue could change the metabolism of the tissue, as well as endocrine and cytokine secretion [6]. On the other hand, adipocytes produce proangiogenic factors and cytokines that can influence vessel remodeling [7].

Cell culture models of vascularized adipose tissue have been explored by several different groups. A general strategy is to co-culture pre-adipocytes or stem cells with endothelial cells in either a 3D material [8] or on 2D surfaces [9,10]. However, a conflict between adipogenic differentiation and vessel formation has been observed in co-culture systems [11]. Treatment with adipogenic induction media results in increased lipid accumulation but may disrupt the formation of vessel structures. In a co-culture model on silk scaffolds, the presence of adipogenic differentiation media resulted in a significant inhibition of endothelial cell survival [12]. To avoid the detrimental effect of adipogenic differentiation media on vessel formation, researchers have proposed to induce the adipogenic differentiation of pre-adipocytes individually before co-culturing with endothelial cells [9,12,13]. After the initial induction, maintenance media may be able to support adipogenic differentiation with a less detrimental effect on endothelial cells [12,14]. However, the co-culture conditions that allow for the simultaneous survival and function of both cell types, including the formation of vascular networks, remain elusive.

The differentiation and function of adipocytes and endothelial cells depend on media supplemented with a variety of factors, including hormones and growth factors. However, there is little to no overlap in the factors used for endothelial cells and adipocytes. Adipogenic factors include insulin, glucocorticoids, and PPAR-γ agonists [14,15], while growth factors such as epidermal growth factor (EGF), basic human fibroblast growth factor (hFGF-B), and vascular endothelial growth factor (VEGF) are often used to induce network assembly and/or the proliferation of endothelial cells [16,17,18]. Commercially available endothelial cell media typically contain supplements to support endothelial cell proliferation, including hydrocortisone, hFGF-B, VEGF, R3-IGF-1, ascorbic acid, hEGF, and heparin. Unfortunately, these same factors may have detrimental effects on the differentiation or function of other cell types. For example, EGF and hydrocortisone have been shown to inhibit adipogenic differentiation and exhibit lipolytic activities [19].

In addition to soluble factors, co-cultureingwith other cell types or in 3D systems can influence cell differentiation and network formation. Three-dimensional culture models are often thought to serve as more accurate mimics of the in vivo environment [20]. Adipose tissue-derived stem cells (ADSCs) cultured in 3D spheroid models exhibited improved differentiation and more robust cell–cell communication compared to cells cultured in 2D [21,22]. For in vitro vessel formation by endothelial cells, supporting cells are often used to provide support directly (cell–cell contact) or indirectly (the production of soluble factors) [23]. Pericytes, fibroblasts, ADSCs, mesenchymal stem cells, and smooth muscle cells have been used as supporting cells and have demonstrated various levels of positive effect on vessel formation [24,25]. Recently, the development of microfluidic platforms has enabled the formation of a perfusable vascular network [26,27]. Vascular networks in 3D micro-constructs flanked with media channels could anastomose with the channels and demonstrate continuous flow within the lumina of the newly formed micro-vessel. This design strategy has been shown to enable control over mass transport in 3D culture [26,28], and has been used to develop different microfluidic models, including microvascular networks [29,30], the blood–brain barrier [31], microtumors [32], and cancer cell extravasation [33].

In this work, the effect of individual media factors on the survival, proliferation, and differentiation of adipocytes and endothelial cells was systematically studied. Two-dimensional culture systems were first used to screen soluble factors in the proliferation of human ADSCs, human umbilical vein endothelial cells (HUVECs), and normal human lung fibroblasts (NHLFs). The media and cellular conditions were then further examined using a 3D spheroid culture of ADSCs. Factors that affect vessel formation were identified using cell suspension in 3D fibrin gel. The combined results of culture conditions were subsequently used to generate a 3D vascularized adipose micro-tissue within a microfluidic device.

## 2. Materials and Methods 

### 2.1. Cell Culture and Differentiation

ADSC were purchased from Lonza (PT-5006, Batch 0000535975). The donor was a 33-year-old male with a body mass index of 25. ADSCs were cultured in DMEM/F12 (11320-033, Gibco, Waltham, MA, USA) supplemented with 10% FBS and 1% antibiotic antimycotic solution (A5955, Sigma, St. Louis, MO, USA), denoted as ADSC-GM. For standard adipogenic differentiation, ADSCs were given ADSC-GM supplemented with 5 µg/mL of insulin, 1 nM of 3,3’,5-triiodoL-thyronine (T3), 2 µg/mL of dexamethasone, and 0.5 mM of isobutylmethylxanthine (IBMX). After 5 days of induction, the cells were given ADSC-GM supplemented with 5 µg/mL of insulin and 1 nM of T3 for the remaining culture time.

HUVECs were purchased from Lonza with pooled donors (C2519A). HUVECs were cultured in the endothelial cell growth medium-2 BulletKit (CC-3162, EGM-2) from Lonza. The EGM-2 kit contains EBM-2 basal medium (CC-3156) and EGM-2 SingleQuots supplements (CC-4176), required for the growth of endothelial cells. 

NHLFs were purchased from Lonza (CC-2512, Lot #: 0000430173). The donor for the cells was a 48-year-old non-smoking male. The doubling time for the NHLFs was around 31 h. The NHLFs were cultured in DMEM (D6429, Sigma) supplemented with 10% FBS and 1% antibiotic antimycotic solution, denoted as NHLF-GM.

### 2.2. Cell Spheroid Formation and Encapsulation

ADSC spheroids were generated by incubating 3000 cells in 150 µL of ADSC-GM with 0.24% methylcellulose in non-tissue culture-treated round-bottom 96-well plates overnight. To encapsulate the cell spheroid, a layer of fibrin was first formed at the bottom of the 48-well plate by adding the mixture of fibrinogen (4 mg/mL) and thrombin (4 U/mL). After the first layer of fibrin was formed, a cell spheroid was placed on the top of the fibrin layer. A second fibrin layer with the same composition was then added to the well to encapsulate the spheroid in the center of the fibrin gel. The well plate was incubated in a 37 °C humidified incubator for 30 min to ensure fibrin polymerization prior to the addition of media to the wells. 

### 2.3. Identification of Supporting Cells for Vessel Formation

HUVECs, ADSCs, and NHLFs were cultured in suspension in fibrin gel to identify the optimal condition for 3D vessel formation. Specifically, 50 μL of fibrinogen (10 mg/mL) and thrombin (6 U/mL) mixture was first added to a 96-well plate to form a layer of fibrin at the bottom. Different combinations and densities of cells were suspended in the same fibrinogen and thrombin mixture (30 μL) and added to the top of the fibrin layer. The well plate was incubated in a 37 °C humidified incubator for 30 min to ensure fibrin polymerization prior to the addition of media to the wells.

### 2.4. Cell Proliferation Assay

The proliferation in different media conditions was evaluated using the CellTiter 96^®^ AQ_ueous_ Non-Radioactive Cell Proliferation Assay (G5430, Promega, Madison, WI, USA) according to the manufacturer’s instructions. The cells were seeded in 96-well plates at 1000 cells/well and incubated in media. After various culture times, 20 μL of combined MTS/PMS solution was added into each well containing 100 μL of fresh cells culture media. The plate was then incubated for 4 h at 37 °C in a humidified, 5% CO_2_ atmosphere. The absorbance at 490 nm was measured using a plate reader (SpectraMax M2, Molecular Devices, San Jose, CA, USA).

### 2.5. Fabrication of a Microfluidic Device

**Figure 1:**
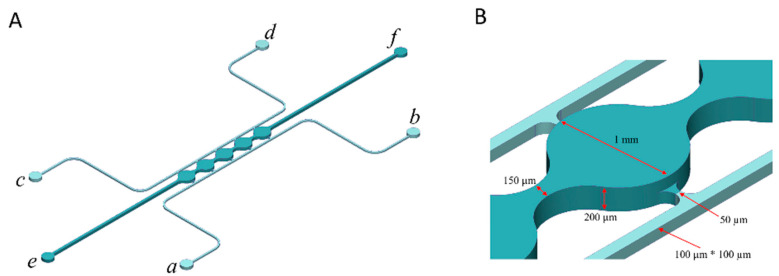
Microfluidic system design and assembly. (**A**) Microfluidic device consists of 5 cell culture chambers (*e*–*f*) flanked by two fluidic channels (port *a–b*, *c–d*). (**B**) Dimensions of the cell culture chamber and fluidic channels.

A microfluidic design that was previously shown to support the formation of a perfusable vascular network was modified to develop the vascularized adipose micro-tissue in this study [26,27]. The microfluidic device consists of cell culture chambers (port *e*–*f*) flanked by two side channels (port *a–b*, *c–d*) (Figure 1A). The cell culture chambers have a height of 200 μm and a width of 1 mm. The side channels are connected on either side of the cell culture chambers via connecting pores with a width of 50 μm and a height of 100 μm (Figure 1B). The microfluidic device was fabricated using polydimethylsiloxane (PDMS) soft lithography. First, a PDMS precursor solution was formed by mixing prepolymer with curing agent at a 10:1 ratio (Sylgard 184, Dow Corning, Midland, MI, USA) and poured onto a computer numerical control (CNC) machined mold. After degassing in a vacuum desiccator for 2 h, the PDMS was cured for 4 h at 65 °C to form a PDMS slab. The PDMS slab was peeled away from the mold and inlet/outlet ports were punched through using a 0.75 mm biopsy punch (World Precision Instruments, Sarasota, FL, USA). The PDMS slab was then bonded to a glass slide to form microfluidic chambers and channels. Plastic vials with the bottoms removed were used as reservoirs and bonded over the ports of fluidic channels (*a*, *b*, *c*, and *d*) on the device using a PDMS precursor solution.

### 2.6. Cell Loading to the Microfluidic Device

For on-chip culture, the cells were loaded into microfluidic devices in a fibrin hydrogel. Briefly, ADSCs, HUVECs, and NHLFs were dissociated with trypsin and counted. The cells were suspended in PBS containing 6 mg/mL of fibrinogen and 6 U/mL of thrombin and loaded into the microfluidic device through the cell chamber inlet (port *e* in Figure 1A), until the solution reached the cell chamber outlet (port *f* in Figure 1A). The loaded device was incubated in a 37 °C humidified incubator for 30 min before the introduction of the co-culture media through the inlets on both sides of the cell culture chambers. Media were added to reservoirs with a height difference to allow flow across the cell culture chambers by hydraulic pressure. The caps of the reservoirs were screwed loosely to allow gas exchange.

### 2.7. Staining

To visualize and confirm the lipid loading, the samples were evaluated with BODIPY staining. First, the cells were fixed with 10% buffered formalin (SF100-4, Fisher Scientific) overnight. After the formalin was removed by flowing PBS through the channels, BODIPY 493/503 (D3922, Invitrogen, Carlsbad, CA, USA) solution in PBS (0.01 mg/mL) then flew through the channels for 3 h before washing with PBS. The samples were imaged using ZEISS Axiovert 200 M confocal microscopy. The quantification of the images was carried out using customized MATLAB scripts.

Immunohistochemical staining for CD31 was performed to visualize the vessel formation. First, the cells were fixed with 10% buffered formalin. After washing with PBS to remove the excess formalin, the cells were permeabilized with 0.5% Triton-X in PBS (PBST) for 10 min. Next, 6% normal goat serum in PBST was applied to the cells to block non-specific antibody binding. Mouse anti-human CD31 primary antibody (clone JC70A, Dako, Glostrup, Denmark, 1:200) was added to the cells in PBST overnight at 4 °C. On the second day, the cells were washed with PBST, goat anti-mouse TRITC secondary antibody was added (NC0208614, JacksonImmuno, West Grove, PA, USA, 1:200), and the mixture incubated for 4 h at room temperature. After staining, the excess antibodies were washed away with PBST. The samples were imaged using a ZEISS Axiovert 200 M confocal microscope. The quantification of the images was carried out using customized MATLAB scripts.

To visualize F-actin, the cells were stained with Alexa Fluor 633 Phalloidin (A22284, Invitrogen, Carlsbad, CA, USA). Specifically, the cells were first fixed with 10% buffered formalin and permeabilized with PBST. Phalloidin was then added to the samples in PBST (1:200) and stained for 1 hour. After washing away the excess phalloidin, the samples were imaged using the ZEISS Axiovert 200 M confocal microscope. The quantification of the images was carried out using customized MATLAB scripts.

### 2.8. Fatty Acid Uptake

The fatty acid uptake by the adipocytes was evaluated after 2 weeks of differentiation using a fluorescently labeled fatty acid analog (BODIPY D-3823, Molecular Probes, Eugene, OR, USA). An amount fo 1 µM of BODIPY D-3823 was added to the media and the samples were cultured for 4 h in the incubator. After incubation, the cells were imaged using the ZEISS Axiovert 200 M confocal microscope. 

### 2.9. Statistics

Statistical analysis was performed using analysis of variance (ANOVA) followed by Tukey’s post-test. In all cases, *p* < 0.05 was considered significant.

## 3. Results

### 3.1. Proliferation

**Figure 2:**
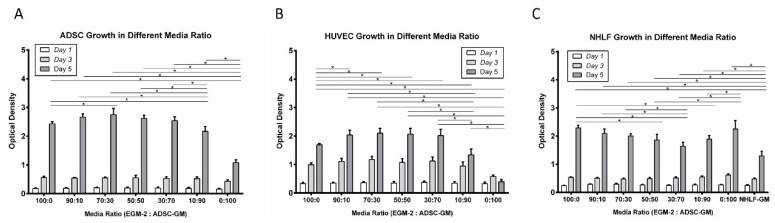
Influence of media composition on cell proliferation. (**A**) ADSC, (**B**) HUVEC, and (**C**) NHLF proliferation in different EGM-2: ADSC-GM media ratios. For all datasets, the values are means ± SD, *n* = 6. One-way ANOVA followed by Tukey’s multiple comparisons test was performed. * Denotes significant difference (*p* < 0.05) between groups.

The two standard endothelial cell and adipocyte culture media, EGM-2 and ADSC-GM, were combined, and the proliferation of HUVECs, ADSCs, and NHLFs was quantified. Interestingly, at days 5 and 7, the ADSCs cultured in 100% EGM-2 media and all mixed media conditions exhibited significantly greater cell proliferation in comparison to the 100% ADSC-GM (Figure 2A). The proliferation of HUVECs was also greater in media ratios from 90:10 to 30:70 (EGM-2: ADSC-GM) relative to 100% EGM-2 (Figure 2B). The HUVECs cultured in high ratios of ADSC-GM, 10:90 and 100%, exhibited significantly less proliferation compared to all the other conditions. The NHLF proliferation was also greater at 5 days in all combinations of EGM-2 and ADSC-GM relative to the standard media conditions (Figure 2C). The collective results indicated that HUVEC, ADSC, and NHLF proliferate well in media mixtures with EGM-2 ratios higher than 30%.

### 3.2. Adipogenic Differentiation

**Figure 3:**
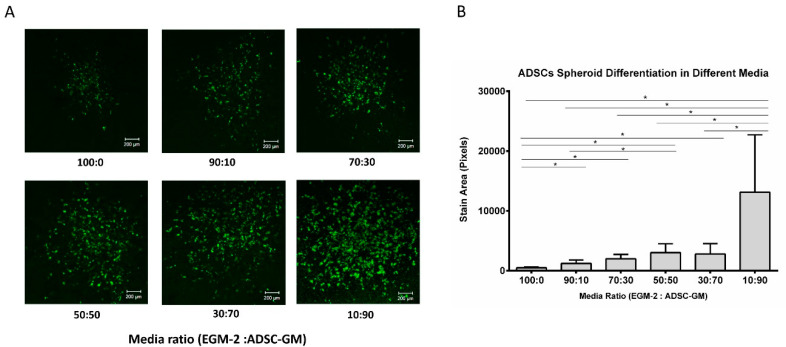
EGM-2 inhibits ADSC spheroid differentiation. (**A**) BODIPY staining of ADSC spheroids in varying media ratios and (**B**) quantitative analysis of BODIPY staining. Values are means ± SD, *n* = 4. One-way ANOVA followed by Tukey’s multiple comparisons test was performed. * Denotes significant difference (*p* < 0.05) between groups.

A three-dimensional spheroid culture system was used to study the influence of media conditions on adipogenic differentiation. ADSC cell spheroids were encapsulated in a fibrin gel and exposed to a differentiation protocol with varying media ratios. First, ADSC spheroids were given adipogenic induction media to initiate differentiation. The induction media contained a basal media with different ratios of EGM-2 and ADSC-GM, 5 µg/mL of insulin, 1 nM of T3, 2 µg/mL of dexamethasone, and 0.5 mM of IBMX. After induction, the spheroids were given maintenance media containing basal media, 5 µg/mL of insulin, and 1 nM of T3. The group with 100% ADSC-GM was not included in this test, since it was shown in the proliferation test that ADSC-GM alone does not support HUVEC survival. After 3 weeks of differentiation, the spheroids were fixed and stained with BODIPY to visualize lipid droplets in the differentiated cells. BODIPY staining was imaged using confocal microscopy (Figure 3A). The staining results showed that the lipid accumulation was lower in media with high EGM-2 levels, suggesting the presence of factors that inhibit adipogenesis in the EGM-2 media (Figure 3B).

### 3.3. Vessel Formation

Vascular network formation in vitro is often enhanced by the presence of support cells [34]. The conditions for generating vascular networks based on varying levels of HUVEC, NHLF, and ADSC (Table 1) were examined first. Cells were suspended in fibrin (10 mg/mL) and cultured in EGM-2 media for 3 weeks. After culture, the samples were stained with CD31 and imaged. The mean vessel length was used to compare the vessel formation.

**Figure 4:**
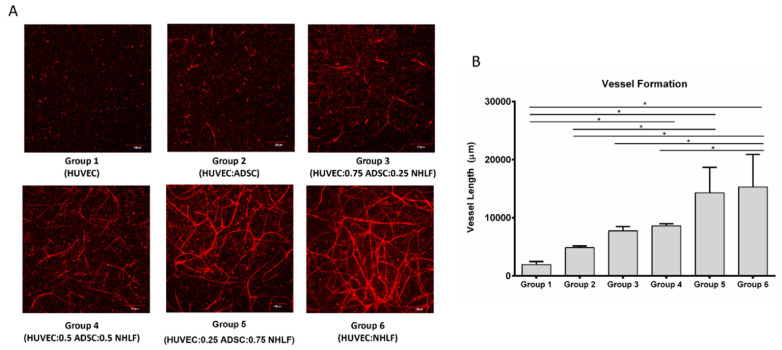
Vessel-like structures in 3D culture. (**A**) CD31 staining of samples from different cell ratio combinations. (**B**) Quantification of total vessel length from different cell ratio combinations. For all datasets, values are mean ± SD, *n* = 4. One-way ANOVA followed by Tukey’s multiple comparisons test was performed. * Denotes significant difference (*p* < 0.05) between groups.

Figure 4A shows the vessel formation from different cell ratio combinations. When HUVECs were cultured alone (group 1), there was little vessel network formation, indicating the need for supporting cells [26]. In group 2 with an equal ratio of ADSCs to HUVECs, only a few vessel-like structures were visible. Visually, the vessel network formation appeared to increase with the NHLFs. The quantification of total vessel length is shown in Figure 4B. Group 4 (0.5 million/mL NHLFs), group 5 (0.75 million/mL NHLFs), and group 6 (1 million/mL NHLFs) had significantly greater mean vessel lengths compared to HUVEC alone. Group 5 (0.75 million/mL NHLFs) had significantly more vessel formation compared to HUVEC and ADSC without NHLF. Group 6 (1 million/mL NHLFs) had significantly more vessel formation compared to group 2 (no NHLFs), group 3 (0.25 million/mL NHLFs), and group 4 (0.5 million/mL NHLFs). The comparison between group 2 (1 million/mL ADSCs) and group 6 (1 million/mL NHLFs) indicates that NHLFs can improve vessel formation more effectively than ADSCs. There was no difference in the mean vessel length between group 5 (0.75 million/mL NHLFs) and group 6 (1 million/mL NHLFs). Group 6 was selected for subsequent study based on its use of a more simple co-culture environment (two cell types) in comparison to group 5 (three cell types).

### 3.4. Vessel Assembly in the Presence of Adipogenic Factors

**Figure 5:**
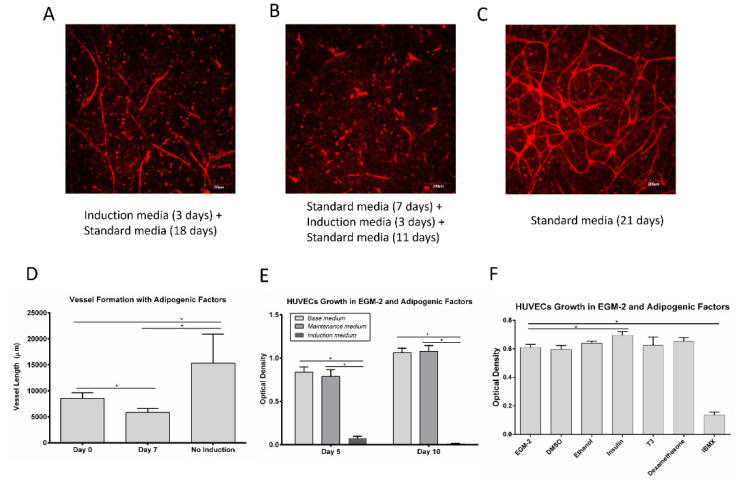
HUVEC vessel network formation (**A–D**) and HUVEC proliferation (**E**,**F**) in the presence of adipogenic factors. (**A**) Network formation with induction media added on day 0 and switched to standard media on day 3. (**B**) Network formation with induction media added on day 7 and switched to standard media on day 10. (**C**) Network formation under standard/control conditions. (**D**) Quantification of mean vessel length in different media conditions. Values are means ± SD, *n* = 4. (**E**) Proliferation of HUVECs in induction media and maintenance media compared to basal media controls. Values are means ± SD, *n* = 5. (**F**) Proliferation of HUVEC with exposure to individual adipogenic factors. Values are means ± SD, *n* = 6. For all the datasets, a one-way ANOVA followed by Tukey’s multiple comparisons test was performed. * Denotes significant difference (*p* < 0.05) between groups.

To test vessel formation in the presence of adipogenic factors, HUVECs (1 million/mL) and NHLFs (1 million/mL) were suspended in fibrin and cultured in well-plates under different conditions for 3 weeks and stained with CD31. The cells were exposed to induction media (EGM-2 supplemented with adipogenic factors) for 3 days, starting at either day 0 or day 7. The exposure of the cells to induction media (Figure 5A,B) hindered the vessel assembly relative to the controls (Figure 5C). The controls showed a densely interconnected network of endothelial cells; exposure to induction media at day 0 resulted in some individual vessels with little interconnectivity, while exposure at day 7 resulted in very few vessel-like structures. The quantification of the vessel length showed that vessel formation was significantly inhibited in the presence of adipogenic factors (Figure 5D).

HUVEC were cultured directly in either the induction media or maintenance media used for adipocytes to examine the impact on proliferation. When examining cell numbers, there was no difference between the culture in the standard HUVEC media and the adipocyte maintenance media at days 5 and day 10 (Figure 5E). However, the cells cultured in adipogenic induction media exhibited significantly lower cell numbers on day 5, with few viable cells left by day 10. This result suggests that some component of the induction media inhibits HUVEC proliferation. To test the effect of individual adipogenic factors on HUVEC proliferation, cells were seeded in 96-well plates with 1000 cells/well and cultured in the presence of adipogenic factors for 5 days. Adipogenic factors were dissolved in DMSO or ethanol to form a stock solution. DMSO (0.2%) and ethanol (0.2%) at these concentrations do not affect HUVEC proliferation (Figure 5F). The proliferation assay results showed that insulin slightly increased the HUVEC proliferation, while IBMX inhibited the HUVEC proliferation (Figure 5F).

### 3.5. Adipogenic Induction of ADSC in a Microfluidic Device

**Figure 6:**
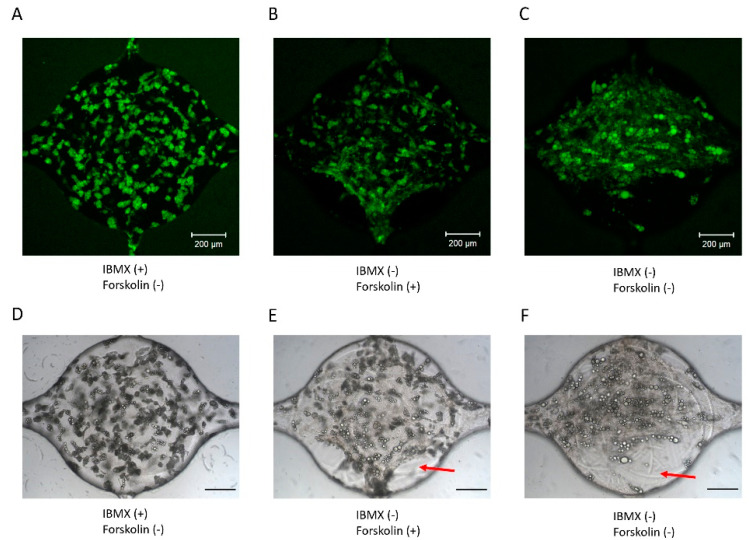
Adipogenic differentiation of ADSC within microfluidic devices. (**A**) BODIPY staining of ADSC differentiated with IBMX or (**B**) forskolin and (**C**) without IBMX or forskolin. (**D**) Brightfield image of ADSC differentiated with IBMX or (**E**) forskolin and (**F**) without IBMX or forskolin.

The above results indicate that IBMX has a detrimental effect on HUVEC vessel formation. To develop the vessel networks and adipocytes together, IBMX would need to be removed from the co-culture media. The next goal was to examine how culture media composition influences adipogenic differentiation. ADSCs were loaded in the microfluidic devices at a density of 5 million/mL in fibrin. Cells were exposed to induction media with or without IBMX. Another cAMP agonist, forskolin, was added to evaluate its potential use as an alternative to IBMX. In contrast to IBMX, forskolin promoted HUVEC proliferation (Figure S1), consistent with previous studies [35]. In all three groups, the cells were given induction media for 5 days, followed by maintenance media for the rest of the culture. After 2 weeks, the cells were fixed and stained with BODIPY to visualize the lipid formation. Images of ADSCs differentiated with IBMX (Figure 6A,D) or forskolin (10 μM, Figure 6B,E) or without cAMP agonist (Figure 6C,F) show that ADSCs differentiate extensively in the presence of IBMX. While lipid droplets were visible, the differentiation appeared reduced in the absence of IBMX. Meanwhile, the fibrin gel structure inside the cell culture chamber was altered and detached from the edges of the chamber (red arrows, Figure 6E,F). The results suggest that IBMX is necessary for the adipogenic differentiation of ADSC and fibrin gel stabilization within microfluidic devices.

### 3.6. Pre-Induction of ADSC for Co-Culture

The above tests revealed a conflict between vessel formation and adipogenic differentiation. IBMX has a detrimental effect on HUVEC proliferation and vessel formation, but at the same time may be necessary for the adipogenic differentiation of ADSCs and fibrin gel stabilization within the microfluidic devices. To address this issue, ADSCs were pre-induced in 2D with adipogenic differentiation media that contains IBMX. After 10 days of differentiation, the cells were dissociated and loaded into the microfluidic devices in fibrin. The cells were given a base media consisting of 50% ADSC-GM and 50% EBM-2, supplemented with insulin, T3, rosiglitazone, and dexamethasone. Combinations of growth factors from the EGM-2 supplement package were added to the culture media to test for the influence of these angiogenic growth factors on adipogenesis (Table 2). After 2 weeks of culture, the cells in the microfluidic devices were stained for lipid accumulation. 

**Figure 7:**
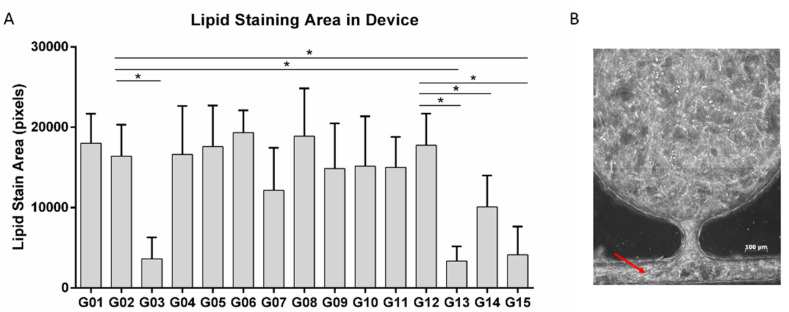
BODIPY staining of differentiated ADSCs in tissue on a chip in the presence of various culture supplements. (**A**) Quantification of lipid staining in the different groups. (**B**) An example of cells migrating into and blocking the side channels (red arrow). For all datasets, the values are mean ± SD, *n* = 5–10. One-way ANOVA followed by Tukey’s multiple comparisons test was performed. *denotes significant difference (*p* < 0.05) between the marked groups.

The quantification of lipid staining is shown in Figure 7A. There were no significant differences between the cells exposed to 100% ADSC-GM (G01) and 50% ADSC-GM and 50% EBM-2 (endothelial cell media without supplements) (G02). Media supplemented with hEGF-B (G03, G13, G15) exhibited significantly reduced lipid levels regardless of the presence of other factors. Hydrocortisone alone did not reduce lipid accumulation (G04); however, a decrease was observed when combined with other factors (G12 and G14). While there were no quantitative differences in lipid levels in the presence of other factors, increased cell migration was observed in the presence of the VEGF (G07) or hFGF-B (G09) blocking of the side channels in the microfluidic system (Figure 7B). Considering these results, hEGF and HC were not included in the media in order to improve the adipogenic differentiation, while VEGF and hFGF-B were not included due to the potential plugging of the microfluidic channels.

After adipogenic differentiation for 10 days, the ADSCs were dissociated and loaded into the microfluidic devices at a density of 5 million/mL. Media formulations supplemented with adipogenic factors were examined for their ability to maintain the differentiated cells (Table 3). After 2 weeks of culture, the cells were stained with lipid and F-actin.

**Figure 8:**
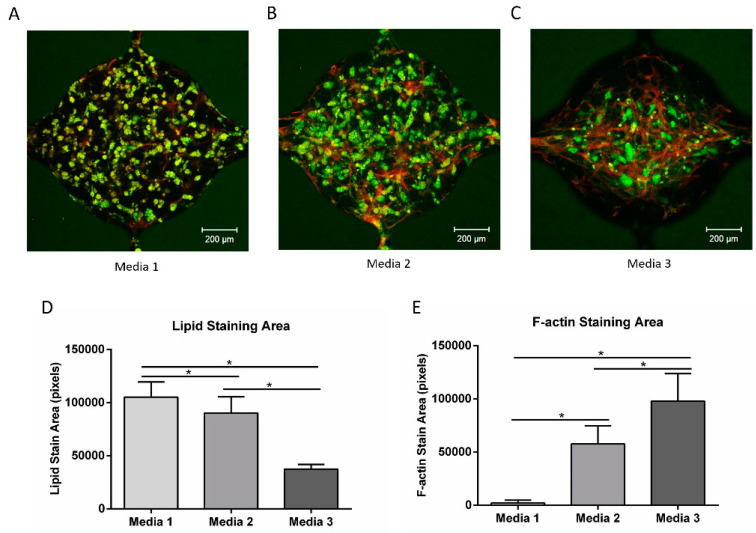
Lipid (BODIPY) and F-actin staining of differentiated ADSCs in different media compositions. (**A–C**) Lipid (green) and F-actin (red) staining of cells cultured in group 1–3 media compositions from Table 3. (**D**) Quantification of the lipid staining area for three groups. (**E**) Quantification of the F-actin staining area for three groups. For all datasets, the values are mean ± SD, *n* = 8–13. One-way ANOVA followed by Tukey’s multiple comparisons test was performed. * Denotes significant difference (*p* < 0.05) between the marked groups.

Figure 8A–C shows the lipid (green) and F-actin (red) staining from the three media. The lipid-loaded cells were distributed throughout the chambers in media 1 (with forskolin, dexamethasone, rosiglitazone) and media 2 (with dexamethasone, rosiglitazone), while the cells in media 3 (without dexamethasone, rosiglitazone, or forskolin) were mostly aggregated near the center of the chamber, with a clear reduction in the overall lipid staining. The quantification of lipid staining revealed increased staining with media 1 compared to the other media (Figure 8D). Significantly greater lipid loading was also observed with the presence of dexamethasone and rosiglitazone (media 2) relative to the control (media 3). The F-actin staining showed an inverse relationship with lipid staining (Figure 8E), suggesting that the partially differentiated ADSCs were going through de-differentiation into fibroblast-like cells [36].

### 3.7. Vascular Network Formation Using Co-Culture Media

**Figure 9:**
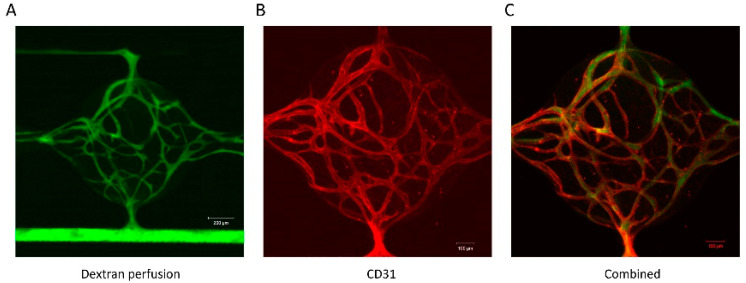
Vascular network formation in the microfluidic device using co-culture media. (**A**) Dextran perfusion through the vessel network. (**B**) CD31 staining of the vessel network. (**C**) Merged image from the dextran perfusion and CD31 staining.

Based on the results described in the previous section, the media 1 combination in Table 3 was considered as potentially optimal for the co-culture of vessel networks and adipocytes in the microfluidic device. To test if this media could support functional vessel network formation in the microfluidic device, HUVECs (2 million/mL) and NHLFs (2 million/mL) were loaded into the cell chambers in fibrin and cultured. At 2 weeks, the function of the networks was tested by perfusing 70 KDa fluorescein isothiocyanate-dextran (FD70S, 25 μg/mL, Sigma) in one microfluidic channel. Figure 9A shows the perfusion of fluorescent dextran in the microfluidic channel and into the microvascular networks. Perfusion can be seen throughout the networks without the escape of the dye from the vessels. Time-lapse images of dextran perfusion through the vessel network (Figure S2) indicated that dextran can diffuse out of the vessel network over time. The diffusion of dextran suggests that the micro-vessels are permeable and could potentially supply nutrients to stromal cells in the interstitium. The sample was subsequently fixed and stained with CD31 to visualize vessel formation (Figure 9B). Figure 9C shows the merged image from the dextran perfusion (green) and CD31 staining (red). The merged image demonstrates that the perfusion was within the microvascular networks. Overall, the results showed that under these newly defined co-culture media conditions, vessel-like networks formed and were able to support media flow.

### 3.8. Vascularized Adipose Tissue in the Microfluidic Device

To form vascularized adipose tissue in the microfluidic device, ADSCs were pre-differentiated using the conditions previously described. ADSCs were first pre-induced in 2D following the standard adipogenic differentiation described in the methods section. The ADSCs were harvested at 10 days and then suspended (5 million/mL) in fibrin with HUVECs (2 million/mL) and supplemented with 0, 1, or 2 million/mL NHLFs before loading into the microfluidic chambers. After 2 weeks of culture, the samples were fixed and stained with CD31 and lipid droplets. 

**Figure 10:**
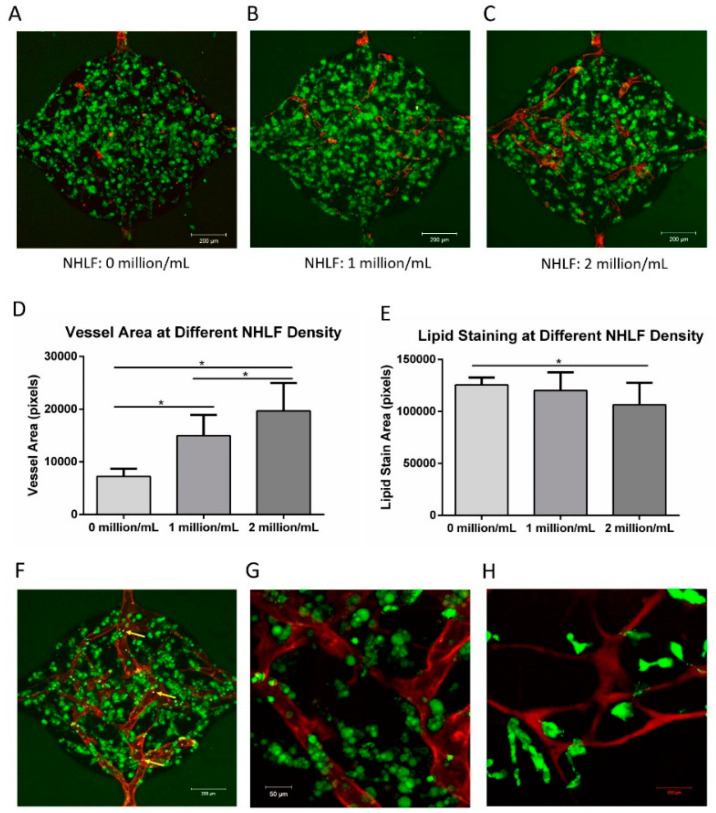
Formation of vascularized adipose tissue. (**A**) Vessel formation and lipid accumulation without NHLF. (**B**) Vessel formation and lipid accumulation with 1 million/mL NHLFs. (**C**) Vessel formation and lipid accumulation with 2 million/mL NHLFs. (**D**) Quantification of vessel formation at different NHLF densities, indicating that vessel formation increases with NHLF. (**E**) Quantification of lipid accumulation at different NHLF densities. (**F**) Microspheres present within the vessel structures (yellow arrows). (**G**) Higher magnification image of (F) stained with CD 31 (red) and lipid droplets (green). (**H**) Dextran perfusion (red) was used to show the functional vessel structures, and perfused fatty acid (green) is taken up by cells in the tissue interstitium. For all the datasets, the values are mean ± SD, *n* = 12–16. One-way ANOVA followed by Tukey’s multiple comparisons test was performed. * Denotes significant difference (*p* < 0.05) between the marked groups.

As observed previously, little network formation was observed in the absence of NHLFs (Figure 10A). However, with both 1 and 2 million cells/mL of NHLF, interconnected vessel networks were present (Figure 10B,C). Quantitatively, the vessel area increased with the increasing NHLF concentration (Figure 10D). This result indicates that NHLFs are vital for HUVEC vessel formation. Qualitatively, lipid loading appears throughout the chambers in all groups. However, quantitative analysis (Figure 10E) shows that there was a slight decrease in the lipid staining area at the maximum concentration of NHLF.

The function of the vessels was examined by adding fluorescent polystyrene microspheres (F13081, Invitrogen) to the media. The microspheres could be seen entering from one channel and passing through the vasculature before exiting through the opposite microfluidic channel (Supplementary Video S1). Figure 10F shows an image of the microspheres within the vessels (blue dots, indicated by yellow arrows). Figure 10G shows a higher magnification image of (F) stained with CD31 (red) and lipid droplets (green). BODIPY D-3823, a fatty acid analog, was injected into the channels to determine if fatty acids supplied by the vasculature would be taken up by the adipocytes into the tissue interstitium. Figure 10H shows that a dextran perfused (red) vessel can be seen in the chamber, while fatty acid uptake (green) was observed by cells near the vessels. The microsphere flow and fluorescein isothiocyanate-dextran perfusion confirmed lumen formation and interconnected networks in the microfluidic network. The fatty acid uptake confirmed that the differentiated adipocytes maintain fundamental functionality in the microfluidic device.

## 4. Discussion

Communication between endothelial cells and adipocytes is critical to adipose development and function. However, endothelial cell–adipocyte co-culture is challenging due to difficulties in identifying a single media condition that will support both cell types. In this work, the media conditions were systematically investigated to determine the effects of various media supplements on the behavior of each individual cell type. 

Endothelial cell culture media is supplemented with a variety of hormones and growth factors, including hEGF, hFGF-B, hydrocortisone, and VEGF. Hydrocortisone and EGF resulted in a significantly reduced lipid accumulation, consistent with a previous report in which they were shown to inhibit adipogenic differentiation [19] and promote the de-differentiation of mature adipocytes [37]. Stromal cells from human adipose tissue exposed to EGF showed a dose- and time-dependent inhibition of adipose cell differentiation [38]. Adipocytes exposed to EGF also exhibit decreased levels of glycerol-3-phosphate dehydrogenase (GPDH), a marker of adipose differentiation, and an increased fibroblast-like cell number [39]. hFGF-B and VEGF in our studies did not inhibit lipid loading but increased proliferation. Others have found that exogenous hFGF-B exerted an inhibitory effect on the lipid droplet accumulation and gene expression of adipogenic markers [40], while VEGF regulates the balance between osteoblast and adipocyte differentiation by mediating the effects of lamin A [41], a suppressor of adipogenesis [42].

A broad range of adipogenic factors (insulin, T3, dexamethasone, rosiglitazone, and forskolin) were added to the co-culture media to support continued differentiation after the partially differentiated ADSCs were loaded into the microfluidic device. The combination of all factors was required to maintain differentiation. Insulin is one of the most widely used adipogenic factors, and has been shown to be sufficient to continue adipogenic differentiation in other systems [14]. Thyroid hormones act as pleiotropic factors by regulating the genes involved in differentiation in many tissues [43], and T3 has been shown to stimulate the adipogenic differentiation of 3T3-F442A cells [44]. However, these factors alone did not sustain adipocyte differentiation in the 3D culture. Forskolin, dexamethasone, and rosiglitazone were also required. In their absence, lipid loading was decreased and the cells exhibited a fibroblastic phenotype. Dexamethasone has multiple, depot-dependent effects on adipocyte gene expression and metabolism that promote central fat deposition [45]. Studies have shown that dexamethasone treatment stimulates the early accumulation of C/EBPβ in primary human preadipocytes [46,47] and downregulates Runx2, a transcription factor that promotes osteogenesis and inhibits adipogenesis [48]. Thiazolidinediones (TZDs) are prescribed for the treatment of type 2 diabetes [49,50]. Rosiglitazone, one of the TZDs, has been shown to stimulate adipogenesis and decrease osteoblastogenesis in human mesenchymal stem cells [51]. Forskolin is a natural compound extracted from the roots of the Indian plant Coleus forskohlii [52]. Forskolin has been clinically used for a variety of conditions, including glaucoma, asthma, and obesity [53]. Similar to IBMX, forskolin increases cellular cAMP and has been shown to effectively accelerate the adipogenic differentiation of 3T3-L1 preadipocytes [54,55].

HUVECs were used to form vessel-like networks in a microfluidic system. For in vitro vascularization, non-endothelial cells are often used as support cells. The support cells can provide support directly (cell–cell contact) or indirectly (the production of soluble factors). Communication between endothelial cells and supporting cells was found to be critical to avoiding network regression and maintaining a stable morphology [34]. The co-culture of HUVECs with ADSCs, MSCs, and NHLFs has been explored and showed that angiogenesis is driven, in part, by the expression of growth factors by the support cells [24]. Human primary fibroblasts have been shown to secrete a variety of growth factors when co-cultured with endothelial cells that can promote vessel formation, including VEGF [56,57]. In the current study, ADSCs and NHLsF were tested for their ability to facilitate vessel formation when co-cultured with HUVECs. The group with NHLFs showed superior vessel network formation compared with ADSCs alone. In the optimized co-culture media, although several pro-angiogenic factors from the EGM-2 media were removed, the culture of HUVECs and NHLFs resulted in an interconnected vascular network within the microfluidic device. Some of the pro-adipogenic factors may also facilitate vessel formation. Thyroid hormones are considered strong pro-angiogenic factors. T3 directly stimulates angiogenesis by promoting endothelial cell proliferation, migration, and capillary network formation [58]. Meanwhile, forskolin has been found to increase endothelial cell proliferation, migration, and tube formation by stimulating typical angiogenic signal events such as NO production and VEGF expression [35].

IBMX was shown to be detrimental to HUVEC survival in this study. IBMX is a cAMP agonist that accelerates the adipogenic differentiation process [54]. IBMX has been shown to be critical for the efficient adipogenic differentiation of human ADSCs in vitro [59]. However, the cAMP elevation induced by IBMX destabilizes endothelial adherens junctions and increases endothelial cell permeability [60]. Exposure to IBMX significantly inhibited HUVEC proliferation and disrupted vessel formation. In an effort to circumvent this issue, researchers reported approaches to differentiate adipogenic cells separately at the beginning and later co-culture with endothelial cells. For example, a porous silk protein scaffold was seeded with HUVECs for 7 days, and then differentiated ADSCs were added for co-culture [12]. In another study, ADSCs were embedded within collagen/alginate microspheres to mimic fat lobules, while HUVECs were later cultured on the surface of the microspheres to mimic micro-vessels in the adipose tissue [10]. In the current study, while lipid accumulation was observed in 3D culture in the microfluidic chamber, the fibrin gel exhibited deformation and degraded under these conditions (Figure 6C,F). When ADSCs were pre-differentiated in 2D in the presence of IBMX prior to loading into a microfluidic chamber, the ADSCs de-differentiated into fibroblast-like cells (Figure 8), leading to fibrin deformation. However, the addition of forskolin to the media facilitated the continued differentiation of ADSC, while facilitating the assembly of vessel networks in this system.

## 5. Conclusions

In this work, co-culture conditions were systematically investigated to identify the potential confounding effects of media supplements on adipocyte differentiation and endothelial cell assembly, with the goal of developing a procedure for engineering a vascularized adipose tissue model. IBMX was detrimental to HUVEC proliferation and vessel formation, while at the same time necessary for maintaining a stable fibrin structure during human ADSC adipogenic differentiation in this study. To circumvent this issue, ADSCs were pre-differentiated in 2D before co-culturing with HUVECs and NHLFs. EGF and hydrocortisone were removed from the co-culture media due to their inhibitory effect on adipogenic differentiation. VEGF and hFGF-B were removed from the co-culture media, since they have shown to promote the outgrowth of cells. Insulin, T3, dexamethasone, rosiglitazone, and forskolin were added to the co-culture media because of their pro-adipogenic properties. This modified condition was used to develop vascularized adipose tissue within a microfluidic device. The vascularized human adipose tissue model could be used as an alternative to animal testing for evaluating new therapies for obesity and metabolic disease.

## Figures and Tables

**Table 1:** Cell combinations for vessel formation.

Groups	HUVEC	ADSC	NHLF
Group 1 (HUVEC)	1 million/mL	0	0
Group 2 (HUVEC:ADSC)	1 million/mL	1 million/mL	0
Group 3 (HUVEC:0.75 ADSC:0.25 NHLF)	1 million/mL	0.75 million/mL	0.25 million/mL
Group 4 (HUVEC:0.5 ADSC:0.5 NHLF)	1 million/mL	0.5 million/mL	0.5 million/mL
Group 5 (HUVEC:0.25 ADSC:0.75 NHLF)	1 million/mL	0.25 million/mL	0.75 million/mL
Group 6 (HUVEC:NHLF)	1 million/mL	0	1 million/mL

**Table 2:** Media compositions for the screening of angiogenic factors.

Groups	Media Composition
G01	ADSC-GM
G02	ADSC-GM + EBM-2
G03	ADSC-GM + EBM-2 + hEGF
G04	ADSC-GM + EBM-2 + Hydrocortisone
G05	ADSC-GM + EBM-2 + AA
G06	ADSC-GM + EBM-2 + Heparin
G07	ADSC-GM + EBM-2 + VEGF
G08	ADSC-GM + EBM-2 + IGF
G09	ADSC-GM + EBM-2 + hFGF-B
G10	ADSC-GM + EBM-2 + VEGF + IGF + hFGF-B
G11	ADSC-GM + EBM-2 + VEGF + IGF + hFGF-B + AA
G12	ADSC-GM + EBM-2 + VEGF + IGF + hFGF-B + AA + Heparin
G13	ADSC-GM + EBM-2 + VEGF + IGF + hFGF-B + AA + Heparin + hEGF
G14	ADSC-GM + EBM-2 + VEGF + IGF + hFGF-B + AA + Heparin + Hydrocortisone
G15	ADSC-GM + EBM-2 + VEGF + IGF + hFGF-B + AA + Heparin + Hegf + Hydrocortisone

**Table 3:** Media groups for the screening of pro-adipogenic factors.

Media	Media Composition	Adipogenic Factors
Media 1	ADSC-GM + EBM-2+IGF + AA + Heparin	Insulin+T3+dexamethasone + rosiglitazone+forskolin
Media 2	ADSC-GM + EBM-2 + IGF + AA + Heparin	Insulin+T3 + dexamethasone + rosiglitazone
Media 3	ADSC-GM + EBM-2 + IGF + AA + Heparin	Insulin + T3

## Supplementary Materials

The following are available online at https://www.mdpi.com/2306-5354/7/3/114/s1, Figure S1: Proliferation of HUVEC in the presence of forskolin, Figure S2: Time-lapse images of dextran perfusion through the vessel network, Video S1: Perfusion of microspheres through vasculature in the microtissue.
