# Supplementary material for: Optimization of Co-Culture Conditions for a Human Vascularized Adipose Tissue Model
Source: Bioengineering (Basel). 2020 Sep 17;7(3):114. doi: 10.3390/bioengineering7030114 (PMC7552791; doi:10.3390/bioengineering7030114)
Supplement: Supplementary file 1 [file bioengineering-07-00114-s001.zip › Supplementary_bioengineering-920765.docx]

Supplementary Information

Optimization of co-culture conditions for a human vascularized adipose tissue model

Feipeng Yang ^1^, Ronald N. Cohen ^2^ and Eric M. Brey ^3,^*

^1^ Illinois Institute of Technology, Department of Biomedical Engineering, Chicago, 60616, USA

^2^ The University of Chicago, Department of Medicine, Chicago, 60637, USA

^3^ University of Texas at San Antonio, Department of Biomedical Engineering and Chemical Engineering, San Antonio, 78249, USA

***** Correspondence: eric.brey@utsa.edu

Figure S1. Proliferation of HUVEC in the presence of forskolin.





**Figure S1.** Proliferation of HUVEC in the presence of forskolin. HUVEC were seeded in 96-well plates at 1,000 cells/well and incubated in EGM-2 supplemented with different concentrations of forskolin (10 μM – 30 μM). At day 5, day 10 and day 15, 20 μl of combined MTS/PMS solution (G5430, Promega) was added into each well containing 100 μl of fresh cells culture media. The plate was then incubated for 4 hours at 37°C in a humidified, 5% CO2 atmosphere. Absorbance at 490 nm was measured using a plate reader (SpectraMax M2, Molecular Devices). For all datasets, values are means ± SD, n = 6. One-way ANOVA followed by Tukey’s multiple comparisons test was performed. *denotes significant difference (p < 0.05) between groups.

Figure S2. Time-lapse images of dextran perfusion through the vessel network.


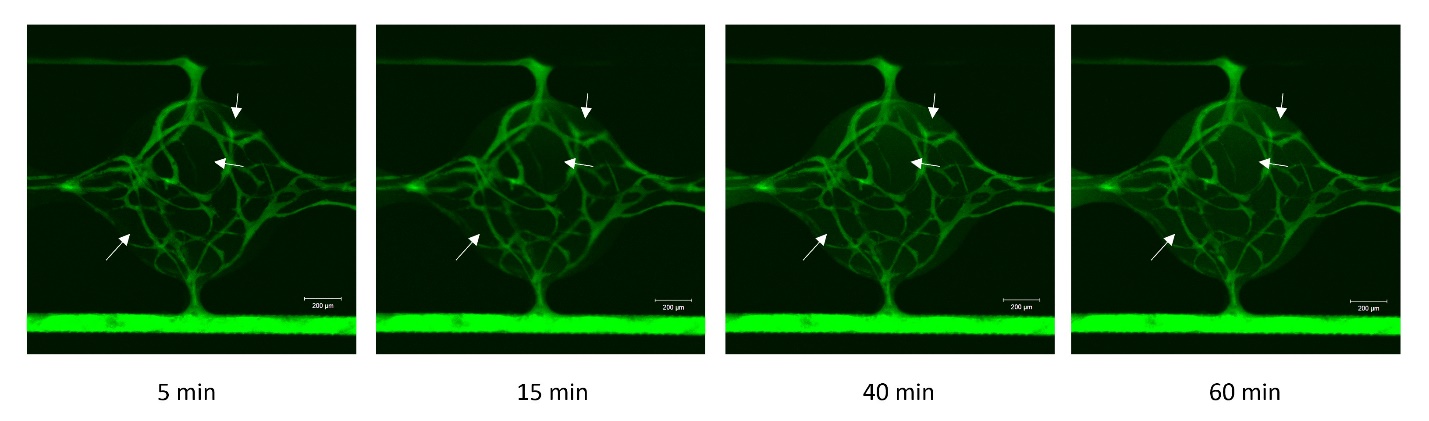


**Figure S2.** Time-lapse images of dextran perfusion through the vessel network. Fluorescence signal in the interstitial space of the vascular network (indicated by white arrows) increases over time, indicating the diffusion of dextran from the vessel structure.
